# The Problems of Nephritis

**Published:** 1910-12-03

**Authors:** 


### The Problems of Nephritis.

The careful study to which the puzzling problems of nephritis have been subjected in recent years is resulting in the establishment of certain more or less assured conclusions on which the complete theory of this intricate lesion will some day be based. During the last five years there has been unceasing activity in following out researches into nephritis, mainly by animal experimentation; and Dr. E. C. Dickson, of San Francisco, sums up, in a recent paper, the present state of knowledge upon points until recently matters of dispute.*

There are four great problems which the purely clinical study of nephritis has failed to solve, and they bear so vitally upon the treatment of the disease, as well as upon its pathology and ætiology, that they are an excellent illustration of the way in which animal experiment forms an aid to clinical study. These four are: The causation of œdema, the relations of the chronic forms of nephritis to the acute; of chronic kidney lesions to the cardio-vascular system; and of kidney lesions to the causation of uræmia.

The first of these, the ætiology of œdema, is both difficult and complicated. As recently as 1904 Dr. Rose Bradford stated that by no known method could an acute parenchymatous nephritis *with œdema* (*i.e.* an acute Bright's disease as it is seen in mankind) be produced experimentally in animals. Since then, however, the use of uranium nitrate to produce this combination has led to many interesting discoveries. It has, in fact, afforded clues to four of the most obscure problems of œdema: (1) Is salt retention or water retention primary? (2) If salt retention is primary, is it due to a true tissue retention, or is it due to a loss of ability of the glomeruli to secrete it? (3) Is salt retention independent of water retention, and are both due to a loss of ability of the renal capillaries to excrete them? (4) Is there any other factor which is necessary for the production of œdema?

The answers which experimental nephritis has yielded to these questions may be stated briefly thus: Water retention is primary, and not secondary, to salt retention. Water and salt retention together are not of themselves sufficient to produce œdema; a further necessary factor is renal injury, however it may be produced. That is, renal injury, vascular injury, and hydræmia are all necessary for the production of œdema.

The relations of the chronic forms of nephritis to the acute are still clouded with a good deal of uncertainty. An explanation of the association of hypertrophy of the heart with chronic kidney lesions is not yet obtained. Quite recently Rautenberg † has by an ingenious method succeeded in causing chronic renal disease with polyuria, high blood-pressure, and progressive cachexia. Whenever life was prolonged for a year or more he found sclerosis of the aorta and sometimes actual aortic aneurysms. From the study of these cases he believes that both of the latter lesions are secondary to high blood-pressure.

Uræmia corresponding to that which terminates so many cases of acute and chronic Bright's disease in man has not been seen in animals. However, a few suggestive and instructive phenomena have been noticed which may indicate the lines of future research. It has been shown that in certain cases of tubal nephritis, to which group uranium and chromate nephritis belong, there may be marked gastro-intestinal disturbance when there is no appreciable decrease in the nitrogenous excretion of the kidneys. It has been supposed, therefore, that the vomiting and diarrhœa of uræmia must be due to some non-nitrogenous substance normally excreted by the tubules of the kidney; and it has been proved that in the urine of normal dogs there is something which, injected intravenously into normal dogs, will lower the blood-pressure, that in the urine of dogs suffering from tubal nephritis this substance is absent, and that in the serum of dogs suffering from tubal nephritis this substance is present. It is not yet determined what this pressor substance is, but it is possible that its vicarious excretion into the bowel accounts for the gastro-intestinal disturbances, and that the same or some similar body is the root cause of the onset of other uræmic symptoms.

* *California State Journal of Medicine,* November 1910.

† *Deutsch. Med. Wochenschrift,* March 24, 1910.

